# Efficacy of Anti-seizure Medications, Quinidine, and Ketogenic Diet Therapy for *KCNT1*-Related Epilepsy and Genotype-Efficacy Correlation Analysis

**DOI:** 10.3389/fneur.2021.834971

**Published:** 2022-01-18

**Authors:** Zehong Lin, Tian Sang, Ying Yang, Yuan Wu, Yan Dong, Taoyun Ji, Yuehua Zhang, Ye Wu, Kai Gao, Yuwu Jiang

**Affiliations:** ^1^Department of Pediatrics, Peking University First Hospital, Beijing, China; ^2^Beijing Key Laboratory of Molecular Diagnosis and Study on Pediatric Genetic Diseases, Beijing, China; ^3^Children Epilepsy Center, Peking University First Hospital, Beijing, China; ^4^Department of Pediatrics, Third Affiliated Hospital of Zhengzhou University, Zhengzhou, China; ^5^Key Laboratory for Neuroscience, Ministry of Education/National Health and Family Planning Commission, Peking University, Beijing, China; ^6^Center of Epilepsy, Beijing Institute for Brain Disorders, Beijing, China

**Keywords:** KCNT1, epilepsy, anti-seizure medications, quinidine, ketogenic diet

## Abstract

**Aim:**

To evaluate the efficacy of anti-seizure medications (ASMs), quinidine, and ketogenic diet therapy (KDT) for *KCNT1*-related epilepsy and to explore genotype-efficacy correlations.

**Methods:**

We collected the data for *KCNT1*-related epilepsy cases from our hospital's medical records and the literature. In total, 50 patients received quinidine, 23 received classical KDT, and 15 received ASMs; all ASM data were from our hospital owing to the lack of detailed ASM data in the literature. The efficacy rates (ERs) of the treatments were compared; an ER that reduced the number of seizures by ≥50% was considered positive. Efficacy according to genotype was also assessed.

**Results:**

The ERs for the 30 patients at our hospital were 40, 26.7, 30, and 44.4% for all treatments, ASMs, quinidine, and KDT, respectively. For all patients (ours and those in previous reports), the overall ERs for quinidine and KDT were 26.0 and 43.5%, respectively (*P* = 0.135). The ERs for quinidine and KDT in functional domain variant-related epilepsy differed significantly (20.6 vs. 53.8%; *P* = 0.037).

**Interpretation:**

KDT may be better at treating *KCNT1*-related epilepsy than quinidine; ASMs were the least effective. KDT is a viable treatment option for functional domain variant-related epilepsy.

## Introduction

*KCNT1* encodes a Na^+^-activated K^+^ channel named Slo2.2, Slack, or KCa4.1. Slo2.2 is the largest known K^+^ channel and is mainly distributed in the frontal cortex of the brain (1). It consists of six transmembrane segments (S1–S6), with a pore domain between S5 and S6 and a cytoplasmic C-terminal domain (2, 3). The C-terminal domain is the largest subunit of the channel, containing regulators of K^+^ conductance (RCKs) and a nicotinamide adenine dinucleotide-binding domain. These subunits exist in the form of tetramers and mediate the channel's allosteric regulation by ligands (2, 3).

Recent studies identified *KCNT1* as a novel driver of pathogenesis in some types of refractory epilepsy (e.g., epilepsy of infancy with migrating focal seizures, autosomal dominant nocturnal frontal lobe epilepsy, West syndrome, Ohtahara syndrome, early myoclonic encephalopathy, focal epilepsy, and multifocal epilepsy) (4–6). However, these diseases are generally highly resistant to traditional anti-seizure medications (ASMs), and the optimal treatment strategy for *KCNT1*-related epilepsy has yet to be determined (5). Because Slo.2.2 is Na^+^-activated, it can be inactivated by Na^+^ blockers such as quinidine. The efficacy of quinidine on *KCNT1*-related epilepsy has been examined in many studies but remains controversial (7–12).

Ketogenic diet therapy (KDT) for refractory epilepsy has recently received increasing attention. The ketogenic diet is a high-fat, low-carbohydrate diet; according to previous reports, it controls seizures by regulating glycolysis, mitochondrial metabolism, neuronal transmission, polyunsaturated fatty acid levels, and other cellular mechanisms (13, 14) and effectively treats *KCNT1*-related epilepsy (5).

Therefore, we hypothesized that KDT would better control *KCNT1*-related epilepsy than do ASMs and quinidine. To test this hypothesis, we compared the efficacy of these treatments by retrospectively analyzing the outcomes of patients with *KCNT1*-related epilepsy at our institution and in the literature. The effect of the *KCNT1* genotype on efficacy was also explored.

Therefore, in this study, we evaluated the efficacy of ASMs, quinidine, and KDT in *KCNT1*-related epilepsy. Further, we explored the efficacy-genotype correlations of *KCNT1*-related epilepsy based on data of our patients and patients reported in the literature.

## Methods

### Participants

We collected 30 patients with KCNT1-related epilepsy treated at our hospital. Detailed clinical data, including age at onset, seizure type, diagnosis, neurologic and cognitive deficits, treatment (ASMs, KDT, quinidine), and genotype, were collected from patient records. This study was approved by the Medical Ethics Committee of Peking University First Hospital (Approval Number: IRB00001052-17030). Written informed consent to participate in this study and for publication of the results was obtained from the guardians of all patients.

**Figure 1:**
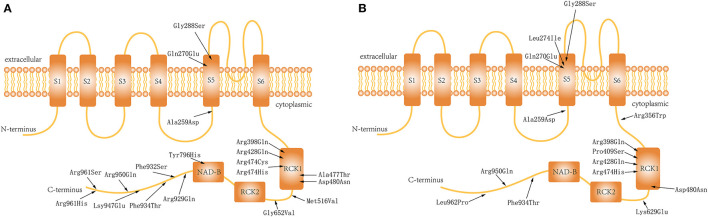
Distribution of the variations in the Slo2.2 channel: **(A)** Patients who received quinidine treatment. **(B)** Patients who underwent ketogenic diet therapy.

We also reviewed all previously published cases in which patients received quinidine (*n* = 40) (5, 12, 15, 16) or KDT (*n* = 14) (5, 12) for treatment of *KCNT1*-related epilepsy, with clear efficacy results. The total number of patients (ours and those in the literature) who received quinidine was 50 (Table 1) and the total number who received KDT was 23 (Table 2). All these patients had received more than at least 3 ASMs before either quinidine or KDT treatment which meaned they were the drug-resistant. Owing to the lack of detailed ASM data in previously reported cases, we used the data from 15 patients from our hospital records who had treated only by ASMs to analyze the efficacy of ASM in KCNT1-related epilepsy. The genotype distribution is shown in the Figure 1, and the analysis of efficacy is shown in Table 3.

**Table 1:** Patients with quinidine treatment (including our patients) (5, 12, 15, 16).

**Variant**	**Case number**	**Efficacy rate**	**Variant**	**Case number**	**Efficacy rate**
c.1193G>A, p.Arg398Gln	5	0	c.1955G>T, p.Gly652Val	1	1/1
c.1238G>A, p.Arg428Gln	6	2/6	c.2386T>C, p.Tyr796His	1	0
c.1420C>T, p.Arg474Cys	1	0	c.2786G>A, p.Arg929Gln	1	0
c.1421G>A, p.Arg474His	7	0	c.2795T>C, p.Phe932Ser	1	1/1
c.1429G>A, p.Ala477Thr	2	1/2	c.2800G>A, p.Ala934Thr	5	1/5
c.1438G>A, p.Asp480Asn	1	0	c.2839A>G, p.Lys947Glu	1	0
c.1546A>G, p.Met516Val	1	0	c.2849G>A, p.Arg950Gln	3	2/3
c.2881C>A, p.Arg961Ser	1	1/1	c.776 C>A, pAla259Asp	1	0
c.2882G>A, p.Arg961His	1	0	c.808C>G, p.Gln270Glu	1	0
c.862G>A, p.Gly288Ser	10	4/10			

**Table 2:** Patients with KDT (including our patients) (5, 12).

**Variant**	**Case number**	**Efficacy rate**	**Variant**	**Case number**	**Efficacy rate**
c.1066C>T,p.Arg356Trp	1	1/1	c.2885T>C,p.Leu962Pro	1	0
c.1193G>A p.Arg398Gln	1	0	c.776 C>A,pAla259Asp	1	0
c.1225C>T,p.Pro409Ser	1	1/1	c.808C>G,p.Gln270Glu	1	0
c.1283G>A,p.Arg428Gln	1	1/1	c.820C>A,p.Leu274Ile	1	1/1
c.1421G>A,p.Arg474His	2	1/2	c.862G>A p.Gly288Ser	5	3/5
c.1438G>A,p.Asp480Asn	1	0	c.2800G>A,p.Ala934Thr	4	1/4
c.1885A>G,p.Lys629Glu	1	0	c.2849G>A,p.Arg950Gln	2	1/2

**Table 3:** Efficacy of quinidine and ketogenic diet in the treatment of KCNT1-related epilepsy.

	**Function domain (S5, RCK, NAD-B)**	**Non-function domain**	***P-*value**
Quinidine	20.6% (7/34)	37.5% (6/16)	0.301
Ketogenic diet	53.8% (7/13)	30% (3/10)	0.402
*P-*value	0.037	1.000	-
	**RCK1**	**Non-RCK1**	***P-*** **value**
Quinidine	13.6% (3/22)	35.7% (10/28)	0.077
Ketogenic diet	50% (3/6)	41.2% (7/17)	1.000
*P-*value	0.091	0.714	-
	**Transmembrane domain**	**Non-transmembrane**	***P-*** **value**
Quinidine	36.4% (4/11)	23.1% (9/39)	0.445
Ketogenic diet	57.1% (4/7)	37.5% (6/16)	0.650
*P-*value	0.630	0.326	-

### Statistical Analysis

Median patient age, age at onset, genetic testing results, disease duration, and the efficacy rates (ERs) for ASMs, quinidine, and KDT were considered as descriptive variables. Fisher's exact test and the chi-square test were used to compare the efficacy of quinidine and KDT in patients stratified and not stratified, respectively, according to the location of the *KCNT1* mutation. Antiepileptic treatment was considered effective if it reduced the occurrence of seizures by ≥50%. *P*-values < 0.05 were considered statistically significant. Statistical analyzes were performed using SPSS for Windows version 26.0 (IBM Corp., Armonk, NY, USA).

## Results

Data were collected from 30 patients treated for *KCNT1*-related epilepsy at our hospital [17 boys and 13 girls; age at onset, 1.25 (0.04–36) months]. All patients had features of developmental and epileptic encephalopathy, and their epilepsy treatments included ASMs, quinidine, and KDT. The ER for all epilepsy treatments was 40%; the ERs for ASMs, quinidine, and KDT were 26.7% (4/15), 30% (3/10), and 44.4% (4/9), respectively. One patient received both KDT and quinidine, which was effective. Clinical data are shown in Table 4.

**Table 4:** Treatments.

**Case**	**Total ASMs tried**	**Quinidine therapy; when; maximum dose (mg/kg.d); effective^a^; drug working time (mo); effective duration (mo)**	**Quinidine side effect**	**KDT; when; effective^a^; KDT working time (mo); effective duration (mo)**	**KDT side effect**
1	TPM, LEV, PB	Not tried	-	Not tried	-
2	VPA, TPM, LEV, PB, OXC, NZP, LCM, CLB^*^	Not tried	-	Not tried	-
3	LEV, OXC, CLB, VPA^*^, CZP^*^	Y; 13 mo after onset; 6; Y; 0.25; 6	emesis	Y; 6 mo after onset; nil; nil; nil	Emesis, constipation
4	TPM, LEV, OXC, CZP, LCM, PB, VPA	Y; 1.5 mo after onset; 12.5; nil; nil; nil	QT internal prolongation	Y; 2 mo after onset; Y 2; 0.5	Emesis, diarrhea
5	TPM, LTG, CBZ, OXC, VGB, CLB, VPA^*^, LEV^*^, CZP^*^	Y; 36 mo after onset; 33; Y; 0.11; 3	None	Not tried	-
6	OXC, VPA, CZP, TPM, LEV, PB	Not tried	-	Not tried	-
7	VPA, LTG, VPA, TPM, LEV, LTG, OXC	Y; 48 mo after onset; 16.7; nil; nil; nil	Abnormal ECG	Y; 36 mo after onset; nil; nil; nil	Constipation
8	VPA, TPM, LTG, CZP	Y; 11 mo after onset; 9; Y; 0.50; 3	Anorexia	Y; 11 mo after onset; Y; 0.50; 3	Feeding difficulties
9	VPA, TPM, LEV, LTG, PB, OXC, Zonisamide, VGB^*^	Y; 17 mo after onset;15; nil; nil; nil	No side effect	Y; 3 mo after onset; nil; nil; nil	Feeding difficulties
10	TPM, LEV, PB	Not tried	-	Not tried	-
11	TPM, LEV, PB	Not tried	-	Not tried	-
12	LEV, ACTH, TPM, LTG, PB	Not tried	-	Not tried	-
13	VPA, TPM, PB, LEV^*^	Y; 5 mo after onset; 7; nil; nil; nil	abnormal ECG	Not tried	-
14	TPM, LTG, CBZ, OXC, CLB, VGB, LCM, VPA^*^, LEV^*^,CZP^*^	Not tried	-	Not tried	-
15	TPM, LEV, PB, OXC, CZP^*^	Y; 3 mo after onset;2; Y; 1; 30;	No side effect	Not tried	-
16	VPA, TPM, PB, LEV	Not tried	-	Not tried	-
17	LEV, TPM, CBZ, PB, OXC, CZP, VGB, CLB, ilepcimide	Not tried	-	Y; NA; nil; nil; nil	NA
18	TPM, LEV, LTG, CBZ, OXC, VPA^*^	Not tried	-	Not tried	-
19	Acetazolamide, LEV, CBZ, VPA, TPM	Y; 2.5 mo after onset; 33; nil; nil; nil	No side effect	Y; 3.5 mo after onset; Y; 3; 26	Low energy
20	TPM, LEV, Acetazolamide, OXC, VPA^*^	Not tried	-	Y; 3 mo after onset; nil; nil; nil	NA
21	VPA, LEV, LTG, OXC, CZP	Y; 60 mo after onset; NA; nil; nil; nil	No side effect	Not tried	-
22	VPA, TPM, LTG, PB, OXC, CZP	Not tried	-	Not tried	-
23	VPA, LEV, CZP, OXC^*^, VGB^*^, CLB^*^	Not tried	-	Not tried	-
24	VPA, LEV, TPM	Not tried	-	Not tried	-
25	LEV, VPA, TPM	Not tried	-	Not tried	-
26	Glucocorticoid, PB, OXC, TPM, VPA	Not tried	-	Not tried	-
27	LEV, OXC, TPM, VPA^*^, VGB^*^	Y; 4 mo after onset; 39; nil; nil; nil	Seizures worsened, QT internal prolongation	Not tried	-
28	LEV, LTG, OXC, VPA, TPM, LCM	Not tried	-	Not tried	-
29	TPM, LEV, LCM^*^, PeraMpanel^*^	Not tried	-	Y; 6 mo after onset; Y; 0.25; 0.25	Feeding difficulties
30	VPA, LEV, OXC, TPM^*^	Not tried	-	Y; NA; Y; NA; NA	NA

a *Effective: Seizure reduction ≥ 50%*.

* *Effective; mo, month; Y, Yes; NA, not applicable; VPA, Valproate; TPM, Topamax; LEV, Levetiracetam; LTG, Lamotrigine; CBZ, Carbamazepine; OXC, Oxcarbazepine; CZP, Clonazepam; NAP, Nitrazepam; PB, Phenobarbital; CLB, Clobazam; VGB, Vigabatrin; LCM, Lacosamide; ACTH, Adrenocorticotropic hormone; KDT, Ketogenic diet therapy; Os, ongoing seizures; ECG, Electrocardiograph*.

Fifty patients, including those at our hospital and in previous reports, received quinidine, with an overall ER of 26.0% (13/50). The ERs for non-functional vs. functional domain variant-related epilepsy (37.5 vs. 20.6%) were not significantly different, nor were those for transmembrane vs. non-transmembrane domain variant-related epilepsy (36.4 vs. 23.1%). The ERs for RCK1 and non-RCK1 domain variant-related epilepsy were 13.6 and 35.7%, respectively, and this difference was of borderline significance (*P* = 0.077).

Twenty-three patients, including those at our hospital and in previous reports, received KDT, with an overall ER of 43.5% (10/23). There was no significant difference in the ERs for non-functional vs. functional (53.8 vs. 30%), RCK1 vs. non-RCK1 (50 vs. 41.2%), or transmembrane vs. non-transmembrane (57.1 vs. and 37.5%) domain variant-related epilepsy.

Finally, we compared the clinical efficacy of quinidine and KDT for treating *KCNT1*-related epilepsy. The overall ER was higher for KDT than for quinidine (43.5 vs. 26.0%; *P* = 0.135), as was the ER for RCK1 domain variant-related epilepsy (50 vs. 13.6%; *P* = 0.091); however, neither difference was significant. The ER for functional domain variant-related epilepsy was significantly higher for KDT than for quinidine (53.8 vs. 20.6%; *P* = 0.037). No significant difference was observed between the ERs for KDT and quinidine for non-functional (30.0 vs. 37.5%; *P* = 1.000), non-RCK1 (41.2 vs. 35.7%; *P* = 0.714), transmembrane (57.1 vs. 36.4%; *P* = 0.630), or non-transmembrane (37.5 vs. 23.1%; *P* = 0.326) domain variant-related epilepsy.

## Discussion

This study compared the efficacy of ASMs, quinidine, and KDT on *KCNT1*-related epilepsy treatment. Our results suggest that KDT may be a more effective treatment than is quinidine, whereas ASMs are the least effective.

Many studies have shown that quinidine effectively inhibits the electrical activity of Slo2.2 channels by blocking Na^+^ influx into cells (17–19); however, its clinical efficacy remains controversial (7–12). In this study, the overall ER for quinidine for *KCNT1*-related epilepsy was ~26.0–30.0%, indicating that not all patients with seizures benefit from quinidine treatment. Because the ER was based on combined quinidine and ASM treatment, we speculate that it may be worse for quinidine alone. Quinidine may indirectly control epilepsy by inhibiting the Na^+^ ion driving effect of the Slo2.2 channel, which reduces Na^+^ permeability. In this study, it was more effective for non-functional than for functional domain variant-related epilepsy, suggesting that mutations in the functional domain directly enhance K^+^ channel activity, and therefore, that the response to Na^+^ blockers was indirect and not obvious.

KDT was formally introduced as an epilepsy treatment in the twentieth century. It has become a commonly accepted treatment for drug-resistant epilepsy owing to its safety and few adverse reactions. In this study, the overall ER for KDT for *KCNT1*-related epilepsy was 43.5–44.4%, which was much higher than that for quinidine and ASMs. According to feedback from the parents of children undergoing KDT, feeding difficulties are the most common problems in the treatment process. Feeding difficulties can directly affect the concentration of ketone bodies in the body; therefore, we believe that the true efficacy of KDT may be higher than the current result.

We found that KDT was more effective in patients with functional vs. non-functional domain-variant related epilepsy, but uniformly effective in patients with other types of domain variant-related epilepsy. Unsaturated free fatty acids have been reported to increase the activity of Ca^+^-activated K^+^ channels, thereby reducing the excitability of neurons (20). Thus, KDT may inhibit Slo2.2 channel activity *via* its effects on the levels of unsaturated fatty acids. Owing to their inhibitory effects on Na^+^ channels, unsaturated fatty acids in ketogenic diets may impede Na^+^ ion binding and the opening of Slo.2.2 channels, thus helping to manage epilepsy (21, 22). The biological mechanism of KDT is complex, multifaceted, and remains unclear; however, it may involve the combined actions of a variety of mechanisms. Hence, KDT potentially has a higher ER for *KCNT1*-related epilepsy.

In this study, KDT seems to have a significantly higher efficacy for *KCNT1*-related epilepsy than did ASMs and quinidine. Compared with quinidine, KDT was equally effective and more effective for non-functional and non-functional domain variant-related epilepsy, respectively. This result suggests that KDT is generally effective for all types of variant-related epilepsy, especially functional domain variant-related epilepsy; therefore, its mechanisms may be diverse. It also supports the use of quinidine for treatment of non-functional domain variant-related epilepsy.

This study had some limitations. Because it was retrospective, some clinical data were missing and the efficacy evaluation process was not always sufficiently detailed. We plan to perform a more detailed analysis in the future. In addition, not all results were statistically significant, although a tendency toward significance was noted in some instances. The lack of significance may reflect the limited number of cases; thus, more cases need to be accumulated to test the validity of our results.

## Conclusion

We evaluated the efficacy of ASMs, KDT, and quinidine for treating *KCNT1*-related epilepsy. KDT may be more effective than is quinidine and thus the more suitable treatment, whereas the efficacy of ASMs was poor. KDT and quinidine confer different therapeutic advantages depending on the *KCNT1* variant: KDT was effective regardless of the variant and especially effective in patients with functional domain variant-related epilepsy, whereas quinidine was effective in patients with non-functional domain variant-related epilepsy. Clarifying the relationship between genotype and curative effect will help optimize treatments and improve outcomes in *KCNT1*-related epilepsy in clinical practice.

## Data Availability Statement

The original contributions presented in the study are included in the article/supplementary material, further inquiries can be directed to the corresponding authors.

## Ethics Statement

The studies involving human participants were reviewed and approved by Medical Ethics Committee of Peking University First Hospital. Written informed consent to participate in this study was provided by the participants' legal guardian/next of kin. Written informed consent was obtained from the individual(s) for the publication of any potentially identifiable images or data included in this article.

## Author Contributions

YJ and KG: study design, analysis, and revision of the manuscript. ZL and YY: follow-up of patient's information. ZL, TS, and YuW: draft preparation. ZL, TJ, YD, YZ, YeW, KG, and YJ: collection of clinical and WES data. All authors contributed to the article and approved the submitted version.

## Funding

This work was supported by the National Natural Science Foundation of China (Grant Numbers: 82171435, 81971211, 12026606, and 81601131), by Beijing Natural Science Foundation (Grant Number: 7212109), by the National Key Research and Development Program of China (Grant Numbers: 2020YFA0804000 and 2020YFA0804003), by the Beijing Key Laboratory of Molecular Diagnosis and Study on Pediatric Genetic Diseases (Grant Number: BZ0317), and by the Fundamental Research Funds for the Central Universities (Grant Numbers: BMU2017JI002, BMU2018XY006, PKU2017LCX06, and BMU2020MX029).

## Conflict of Interest

The authors declare that the research was conducted in the absence of any commercial or financial relationships that could be construed as a potential conflict of interest.

## Publisher's Note

All claims expressed in this article are solely those of the authors and do not necessarily represent those of their affiliated organizations, or those of the publisher, the editors and the reviewers. Any product that may be evaluated in this article, or claim that may be made by its manufacturer, is not guaranteed or endorsed by the publisher.

## Abbreviations

ASM: anti-seizure medication
ER: efficacy rate
KDT: ketogenic diet therapy
RCK: regulator of K^+^ conductance.
